# Contribution of Global Polio Eradication Initiative–Funded Personnel to the Strengthening of Routine Immunization Programs in the 10 Focus Countries of the Polio Eradication and Endgame Strategic Plan

**DOI:** 10.1093/infdis/jiw567

**Published:** 2017-06-30

**Authors:** Maya M. V. X. van den Ent, Rachel D. Swift, Sameer Anaokar, Lea Anne Hegg, Rudolf Eggers, Stephen L. Cochi

**Affiliations:** 1 UNICEF Programme Division, United Nations Children’s Fund, New York, New York;; 2 Boston Consulting Group, Los Angeles, California;; 3 Global Development, Bill and Melinda Gates Foundation, Seattle, Washington;; 4 Global Immunization Division, Centers for Disease Control and Prevention, Atlanta, Georgia; and; 5 Department of Immunization, Vaccines, and Biologicals, World Health Organization, Geneva, Switzerland

**Keywords:** Poliomyelitis, eradication, endgame, routine immunization, health systems.

## Abstract

**Background.:**

The Polio Eradication and Endgame Strategic Plan (PEESP) established a target that at least 50% of the time of personnel receiving funding from the Global Polio Eradication Initiative (GPEI) for polio eradication activities (hereafter, “GPEI-funded personnel”) should be dedicated to the strengthening of immunization systems. This article describes the self-reported profile of how GPEI-funded personnel allocate their time toward immunization goals and activities beyond those associated with polio, the training they have received to conduct tasks to strengthen routine immunization systems, and the type of tasks they have conducted.

**Methods.:**

A survey of approximately 1000 field managers of frontline GPEI-funded personnel was conducted by Boston Consulting Group in the 10 focus countries of the PEESP during 2 phases, in 2013 and 2014, to determine time allocation among frontline staff. Country-specific reports on the training of GPEI-funded personnel were reviewed, and an analysis of the types of tasks that were reported was conducted.

**Results.:**

A total of 467 managers responded to the survey. Forty-seven percent of the time (range, 23%–61%) of GPEI-funded personnel was dedicated to tasks related to strengthening immunization programs, other than polio eradication. Less time was spent on polio-associated activities in countries that had already interrupted wild poliovirus (WPV) transmission, compared with findings for WPV-endemic countries. All countries conducted periodic trainings of the GPEI-funded personnel. The types of non–polio-related tasks performed by GPEI-funded personnel varied among countries and included surveillance, microplanning, newborn registration and defaulter tracing, monitoring of routine immunization activities, and support of district immunization task teams, as well as promotion of health behaviors, such as clean-water use and good hygiene and sanitation practices.

**Conclusion.:**

In all countries, GPEI-funded personnel perform critical tasks in the strengthening of routine immunization programs and the control of measles and rubella. In certain countries with very weak immunization systems, GPEI-funded personnel provide critical support for the immunization programs, and sudden discontinuation of their employment would potentially disrupt the immunization programs in their countries and create a setback in capacity and effectiveness that would put children at higher risk for vaccine-preventable diseases.

The Polio Eradication and Endgame Strategic Plan (PEESP) defined its second objective around the strengthening of immunization systems and established a target that at least 50% of the time of field personnel funded by international partners of the Global Polio Eradication Initiative (GPEI; hereafter, “GPEI-funded personnel”) should be dedicated to the strengthening of immunization systems; additional components of the second objective involve increasing coverage with the third-dose of diphtheria, tetanus, and pertussis containing vaccine (DTP3) in districts where the risk of polio is high, introducing inactivated poliovirus vaccine, and withdrawing oral polio vaccines following certification of eradication of the wild type virus [1]. This is in line with the Global Vaccine Action Plan, which aims to achieve 90% coverage for all antigens nationally and at least 80% coverage in every district [2], and the Measles and Rubella Strategic Plan 2012–2020, which targets 95% coverage with measles vaccine in every district during supplementary immunization activities [3].

Because wild poliovirus has been endemic until recently in many countries with very weak health systems, significant investments have been made by GPEI to increase human-resources capacity at national levels and in high-risk polio districts to achieve the eradication goal. In 2012, close to 28 000 GPEI-funded personnel were active in the 10 focus countries of the PEESP (Table 1) These personnel have primarily been funded and employed by GPEI international partners and have historically focused on providing technical and programmatic support to improve polio campaign quality and surveillance. Increasingly, GPEI-funded personnel have contributed to the support of wider immunization and mother-and-child-health programs. For example, since 2011 in India, GPEI-funded personnel have been monitoring routine immunization (RI) sessions.

**Table 1.** Summary of Time Allotments of Global Polio Eradication Initiative–Funded Personnel, by Priority Area, Training, and Inclusion of Routine Immunization (RI) in Terms of Reference

Variable	WHO African Region						WHO Eastern Mediterranean Region			WHO South Asian Region	
	Angola	Chad	DRC	Ethiopia	Nigeria	South Sudan	Afghanistan	Pakistan	Somalia	India	Overall
GPEI-funded staff in 2012, no.	121	171	199	130	11,181	390	3,198	2,598	212	9,743	27,943
GPEI-funded managers, no.	23	15	NA	NA	489	32	48	280	NA	NA	>1000
Respondents, no.	19	8	24	7	173	5	30	86	9	106	467
Activity, time allocated, %											
Polio eradication	29.0	40.4	38.9	44.1	36.7	26.8	74.2	64.4	54.1	43.8	46
RI	26.0	32.4	23.1	17.1	21.8	15.2	15.8	18.4	18.4	27.8	22
Measles and rubella control	12.7	7.3	13.7	20.9	6.8	14.4	3.5	5.5	11.1	11.2	8
New vaccine introduction	8.9	1.4	3.6	0.7	4.4	8.8	1.1	2.2	0.9	4.1	4
Child health days or weeks	4.2	0.0	2.3	1.4	7.0	4.0	0.9	1.7	2.8	0.8	4
Maternal, newborn, and child health and nutrition programs	3.7	2.4	2.4	2.1	8.7	2.6	0.8	1.7	7.2	2.0	5
Health systems strengthening	5.4	3.8	5.6	4.4	4.3	5.0	0.5	2.3	1.1	4.4	4
Immunization-related activities beyond polio	61	47	51	47	53	50	23	32	42	50	47
Sanitation and hygiene	2.8	0.5	1.9	0.7	4.2	0.0	2.0	1.2	0.4	0.9	2
Natural disasters and humanitarian crises	2.3	7.1	3.0	2.9	1.6	16.0	0.7	0.5	2.8	0.4	1
Other diseases or program areas	5.0	4.9	5.4	5.6	4.6	7.2	0.5	2.0	1.1	4.6	4
Personnel formally trained in RI, %	95	100	75	86	87	60	25	65	88	88	
Personnel who have RI included in ToR, %	100	100	96	43	97	60	96	84	88	92	
Personnel who included training and RI in ToR, no.	19	8	24	7	156	5	24	49	8	103	403
DTP3 coverage, %^a^											
In 2013	77	48	74	72	46	45	70	72	42	83	
In 2015	64	55	81	86	56	31	78	72	42	87	

Abbreviations: DRC, Democratic Republic of the Congo; DTP3, diphtheria, tetanus, and pertussis vaccine; NA, not available; ToR, terms of reference.

^a^Data are from [10].

In their assessment of the impact of polio eradication activities on RI programs, Closser et al [4, 5] concluded that there was no compelling evidence of the effects—positive or negative—of polio eradication campaigns. Impact was largely dependent on the context. In settings with strong leadership and few campaigns, such as Rwanda and Ethiopia, Closser et al found that polio eradication activities had a positive impact on RI programs. In areas where there was civic unrest, weak health systems, and ongoing polio transmission, as in Nigeria and Pakistan, polio eradication activities did not contribute to the improvement of health systems, but there was no evidence that eradication activities worsened these systems either. Health worker motivation in settings with many (ie, >4) polio campaigns each year was detrimental. Allocation of time among government staff to polio eradication activities accorded with national priorities, but the authors reported that this reduced the time dedicated to RI activities and primary health care. More recently, focused approaches have been reported to take the opportunity to use assets associated with polio eradication to strengthen immunization systems and primary health care. Elsewhere in this supplement, there are extensive reports on how polio-related assets strengthened broader immunization goals [6–8].

Until recently, there had not been a formal assessment on the amount of time that GPEI-funded personnel spent on broader immunization and mother-and-child-health goals. This article describes the self-reported profile of how GPEI-funded personnel allocate their time toward immunization goals and activities beyond those associated with polio eradication, the training they have received to conduct tasks to strengthen RI systems, and the type of tasks they have conducted. 

## METHODS

A survey was conducted among about 1000 managers of GPEI-funded personnel involved in frontline polio eradication activities, to develop a quantitative view of the proportion of time frontline staff spend on polio eradication, RI, and other health and development priorities. The survey collected information on broader issues, such as the criticality of polio eradication programs to other health priorities, the impact of these programs on broader immunization goals, and the capabilities that could be at risk if polio funding and support for personnel was reduced [9].

Respondents were asked to approximate the amount of time spent activities across 10 categories (equaling 100%). The first category (polio eradication activities) addressed support for the following polio eradication efforts: policy and strategy development, planning, management and oversight, implementation and service delivery, communications and community engagement, disease surveillance and data analysis, and partnership and coordination. The second (RI) targeted activities related to strengthening local RI systems, such as Expanded Program on Immunization (EPI) capacity building, monitoring and supervision of immunization sessions, data management and analysis, and design and implementation of communication strategies for RI. The third (measles and rubella) focused on activities related to targeted measles and rubella prevention and elimination, including vaccination campaign support and case surveillance. The fourth category (new vaccine introduction) consisted of activities related to facilitating the introduction of new and underutilized vaccines, such as pentavalent vaccine (DTP, *Haemophilus influenza* type B, and hepatitis B), pneumococcal conjugate vaccine, and rotavirus vaccine; the second dose of measles vaccine; development of proposals for new-vaccine introduction; and support provide to governments with respect to readiness assessment and planning, development of operational guidelines, postintroduction evaluations, and surveillance. The fifth (child health days or weeks) targeted activities related to creating awareness around issues of child health, including advocacy with political, administrative, religious, and community leaders; assistance with health education; provision of basic health services to children; and implementation of child health days. The sixth category included activities related to improving sanitation and hygiene, such as social mobilization to sensitize communities on importance of sanitation and hygiene, supporting water quality assessment and data gathering, assistance with education on waste disposal and infection control, and supporting installation of toilets in schools. The seventh focused on activities to counsel and educate pregnant women and their families, including communication on exclusive breast feeding and importance of institutional delivery; assistance with policy development, program evaluation, and high-level advocacy; supporting distribution of vitamins; and monitoring of nutritional support for malnourished children. The eighth targeted activities related to improving overall healthcare systems and infrastructure, such as provision of data to governments to improve services; assistance with development of government strategy and long-term planning; supporting the overall supply chain; strengthening partnerships among donors, nongovernmental organizations, and governments; and surveillance for other diseases. The ninth comprised activities related to the response to disease outbreaks and other crises and disasters, such as floods, landslides, earthquakes, and humanitarian crises. The tenth was composed of other diseases or program areas that were not included in the first 9 categories.

The survey was distributed internally by participating agencies and was open for 8 weeks. The World Health Organization (WHO), the United Nations Children’s Fund (UNICEF), the Centers for Disease Control and Prevention, and Rotary International were tasked with distributing the survey link via email to their respective country program leaders across the 10 countries, who then identified the managers of frontline staff. The managers completed the survey on behalf of their teams. The number of surveys to managers of frontline workers distributed is presented in Table 1 for 6 focus countries. For the remaining 4 focus countries, we estimated the number of managers to be >200 in the 4 countries, but no exact numbers were available because personnel numbers changed rapidly over time. Only completed or nearly completed surveys were included in the final analysis. Surveys deemed inadequate included those for which only demographic information was entered or incomplete allocation of personnel time was reported.

Alongside the survey, Boston Consulting Group conducted interviews with country-level leadership not only to validate insight from the survey, but to better understand reasons for success or failure in supporting other health priorities, particularly with regard to meeting or exceeding targets for RI support. The interviews were free-flowing discussions and played an important part in contextualizing the situation and options for moving forward in each country.

## RESULTS

**Figure 1.**
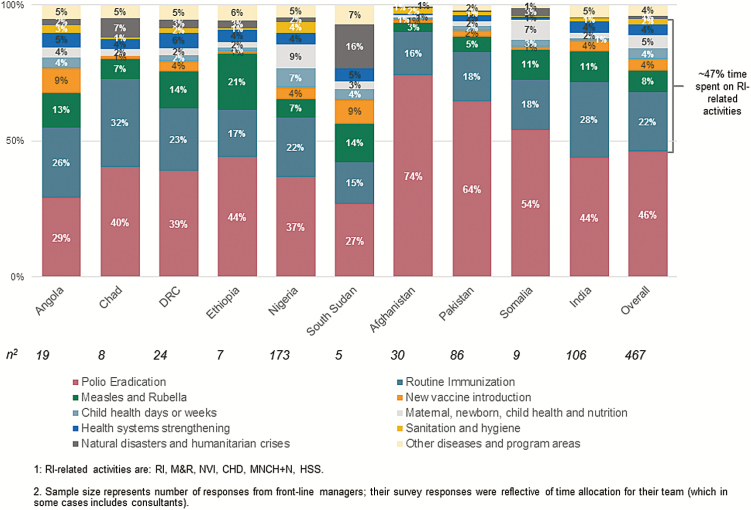
Allocation of personnel time in 10 focus countries of the Polio Eradication and Endgame Strategic Plan. Mean personnel time allocated to routine immunization (RI) was approximately 22%, and mean personnel time allocated to RI-related activities (ie, RI; measles and rubella control; new vaccine introduction; child health days or weeks; maternal, newborn, and child health and nutrition programs; and health systems strengthening) was approximately 47%. Sample sizes represents the number of responses from frontline managers; their survey responses were reflective of time allocation for their team (which, in some cases, includes consultants). DRC, Democratic Republic of the Congo.

From the 10 priority countries, responses from 467 GPEI-funded personnel were included in the analysis (Table 1). GPEI-funded frontline workers spent on average approximately 22% of their time on RI across all focus countries, approximately 8% on measles and rubella control activities, and approximately 47% on immunization goals and activities beyond polio (Figure 1).

The time spent on RI activities, by country, ranged from 15% to 32% (15% in South Sudan, 16% in Afghanistan, 17% in Ethiopia, 18% in Somalia, 18% in Pakistan, 22% in Nigeria, 23% in the Democratic Republic of the Congo [DRC], 26% in Angola, 28% in India, and 32% in Chad). WHO and UNICEF personnel allocated a similar proportion of time to RI (23% and 21%, respectively). The program area with the next-highest time allocation was measles and rubella control (average, 8%; range, 4%–21%), with values of 3% in Afghanistan, 5% in Pakistan, 7% in Nigeria, 7% in Chad, 11% in Somalia, 11% in India, 13% in Angola, 14% in the DRC, 14% in South Sudan, and 21% in Ethiopia.

There was variation in time allocation across countries. Countries in the WHO Eastern Mediterranean Region (EMR) countries—particularly the polio-endemic countries of Afghanistan and Pakistan—spend substantially more time on polio eradication activities (74% and 64%, respectively) and comparatively less time supporting other health priorities (Table 1 and Figure 1). Countries where polio is not endemic and those where transmission has been interrupted (including Nigeria at the time of the survey) spend less time on polio eradication activities, allowing personnel to spend a greater portion of their time on other health and development priorities, most notably RI strengthening and measles and rubella control.

**Figure 2.**
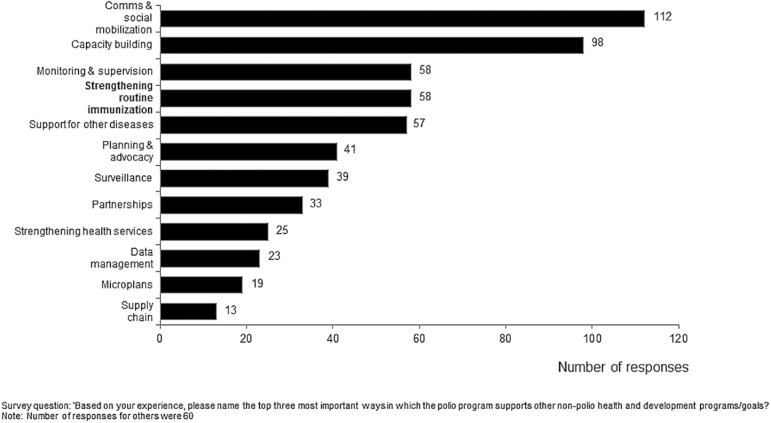
Polio staff perceived that strengthening routine immunization programs is one of the Global Polio Eradication Program’s most significant impacts on non–polio-related health priorities. Data are based on responses to the following survey question: “Based on your experience, please name the top three most important ways in which the polio program supports other non-polio health and development programs/goals?” The number of responses citing activities other than those listed was 60.

GPEI-funded staff were asked to rank their views of the most important influences of the polio eradication program on non–polio-related health and development programs and goals. The top 5 program activities selected (in rank order) were communication and social mobilization, capacity building, monitoring and supervision, strengthening RI, and support for control of other diseases, such as measles and rubella (Figure 2).

**Figure 3.**
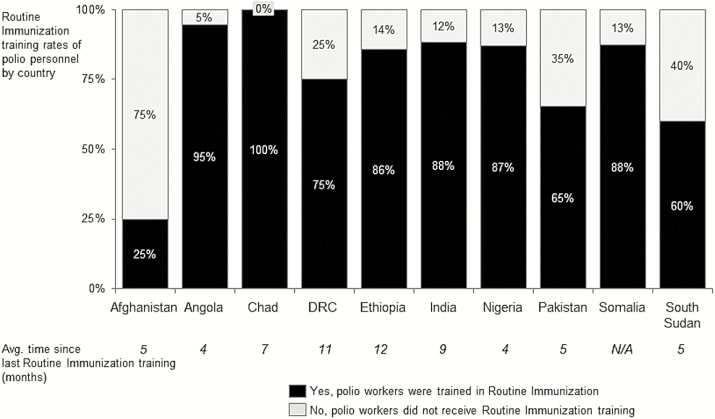
Routine immunization (RI) training rates among Global Polio Eradication Initiative–funded personnel, by country, with average time since last RI training session. DRC, Democratic Republic of the Congo; NA, not applicable.

Countries with higher RI training rates reported significantly higher RI time allocation (r = 67%; *P* < .025). The same relationship holds true for RI-related activities (r = 78%; *P* < .005; Figure 3). This relationship was substantiated through country leadership discussions. It was noted that, while inclusion of RI in terms of reference is necessary, reinforcing the importance of RI strengthening and introducing new tools and approaches to personnel was critical to keeping RI in focus and a top priority.

In the interviews with immunization managers, a lower frequency of campaigns, sufficient quantity and quality of personnel, strong government commitment, a high level of transparency, and effective accountability mechanisms were commonly mentioned as critical to increasing the time spent on RI and program improvements.

## DISCUSSION

The survey has several limitations. The survey was completed by frontline managers, which may have introduced a bias. This was done for 2 reasons. First, targeting frontline managers provided the best balance between data fidelity and efficiency. They are closer than country leadership to the activities on the ground and are of a manageable sample size (approximately 1000), compared with the number of frontline workers (close to 29 000), making it logistically feasible under the challenging circumstances of conducting this field survey to achieve a response rate that is high and representative of the universe of frontline workers. Second, some frontline staff may not be able to reliably access an online survey or may not read English (the only language the survey was coded in), which could therefore reduce the response rate and data confidence.

The exact number of surveys distributed was reported for 6 of 10 countries. In the 4 remaining countries, the survey was distributed to about 200 managers.

Some country and agency data are more robust than others. In particular, the number of responses and yield associated with UNICEF personnel managers for South Sudan and Pakistan are quite low, despite reminders sent to the managers. In these 2 instances in particular, we caution drawing broad implications from the survey results, given the sample size and yield.

GPEI-funded personnel spent substantial time on broader immunization and mother and child health program activities. While the amount of time varied by country, GPEI-funded personnel were being used to improve RI program activities and strengthen systems, control measles and rubella, conduct surveillance activities, and support broader maternal, newborn, and child health programs. This is particularly true in countries where wild poliovirus circulation had been interrupted and the number of campaigns was reduced. This finding is in line with findings reported by Closser et al [5].

Analysis of the type of activities conducted in RI reveals that, across regions, GPEI-funded personnel were most frequently engaged in advocacy, communications, and community engagement, followed by research, which included activities related to learning and refining RI approaches in the field, and special studies. Notably, training and capacity building was a top activity in India and less so in the other regions. Personnel terms of reference included RI as per survey data, demonstrating the increasing formal role of GPEI-funded staff in RI activities. However, RI training rates are quite variable—in Chad, all GPEI-funded personnel (100%) have been trained in RI. In contrast, only 56% of surveyed personnel in the EMR countries (Afghanistan, Pakistan, Somalia) have received RI training. In Chad, the revitalization of the RI programs depend largely on GPEI-funded staff both at national level and decentralized levels.

GPEI-funded assets have helped raise awareness of the importance of RI for polio and other vaccine-preventable diseases, yield data and surveillance information that allow countries to develop evidence informed RI planning and implementation, facilitate systematic RI microplanning, provide RI services to deprived communities, such as nomads, migrants, rural remote populations [6]. Sudden withdrawal of GPEI-funded personnel could therefore have a negative impact on a number of health system strengthening activities outside of polio eradication. As reported by the respondents, the largest impact would be a decrease in RI coverage rates and its associated effects, including higher dropout rates, less community awareness of RI activities, and decreased monitoring of RI programs and training and supervision of RI personnel.

While self-reported data by GPEI frontline managers may introduce some bias, it is clear that the polio eradication program has been successful in increasing capacity at the local level in countries and places where systems are weakest—districts where the risk of polio is high. Decentralizing support to RI microplanning, and conducting district taskforce meetings to analyze data for action are promising approaches to improve the program, as shown in India. In Nigeria and India, GPEI has shifted its social mobilization efforts from being externally driven to local agents, often women, who promote immunization, child health, and birth registration in their own communities, with tangible results and impacts.

Although a direct causal relationship between national RI improvements and GPEI-funded personnel’s’ time spent on non–polio eradication activities is challenging to establish, in 6 of 10 focus countries (Chad, the DRC, Ethiopia, Nigeria, Afghanistan, and India), DTP3 coverage improved between 2013 and 2015; in 2 (Somalia and Pakistan), no progress was reported; and in 2 (Angola and South Sudan), coverage regressed between 2013 and 2015, based on WHO and UNICEF estimates for immunization coverage [10]. The contributions of GPEI-funded personnel need to be valued by governments, programs, and donors, to guide the transition of polio assets and infrastructure to sustain the essential functions that will be needed to continue to strengthen systems, improve RI activities, control measles and rubella, and promote the health of these most disadvantaged communities, which were previously affected by polio. Therefore, polio transition planning is critical in taking the findings of this study and developing a business case for transitioning polio assets to build more equitable and stronger health and immunization systems.

## Notes

***Disclaimer.*** The findings, interpretations, and conclusions expressed in this article are those of the authors and do not necessarily reflect the policies or views of the organizations to whom the authors are affiliated.

***Financial support.*** This work was supported by the Bill and Melinda Gates Foundation.

***Supplement sponsorship.*** This work is part of a supplement coordinated by the Task Force for Global Health with funding provided by The Bill and Melinda Gates Foundation and the Centers for Disease Control and Prevention.

***Potential conflicts of interest.*** All authors: No reported conflicts. All authors have submitted the ICMJE Form for Potential Conflicts of Interest. Conflicts that the editors consider relevant to the content of the manuscript have been disclosed.

## References

[1] World Health Organization (WHO). Polio Eradication & Endgame Strategic Plan 2013—2018. Geneva: WHO, 2013.

[2] World Health Organization (WHO). Global Vaccine Action Plan 2011–2020. Geneva: WHO, 2013.

[3] World Health Organization (WHO). Measles and Rubella Strategic Plan: 2012–2020. Geneva: WHO, 2012 http://apps.who.int/iris/bitstream/10665/44855/1/9789241503396_eng.pdf Accessed 31 October 2016.

[4] Closser S, Rosenthal A, Parris T Methods for evaluating the impact of vertical programs on health systems: protocol for a study on the impact of the global polio eradication initiative on strengthening routine immunization and primary health care. BMC Public Health 2012; 12:728. 22938708 10.1186/1471-2458-12-728 PMC3499151

[5] Closser S, Cox K, Parris TM The impact of polio eradication on routine immunization and primary health care: a mixed-methods study. J Infect Dis 2014; 210 (Suppl 1:S504–13. 24690667 10.1093/infdis/jit232 PMC4197907

[6] van den Ent MMVX, Mallya A, Sandhu H Experiences and lessons from polio eradication applied to immunization in ten focus countries of the polio eradication endgame strategic plan. J Infect Dis 2017; 216 (suppl 1):S250–9. 10.1093/infdis/jix047 PMC5853381 28838187

[7] Yared G Y, Pascal M, Alex G, Tasfaye E Strategic engagement of technical surge capacity for intensified polio eradication initiative in Nigeria, 2012–2015. J Infect Dis 2016; 213:S166–23. 10.1093/infdis/jiv494 PMC4818549 26912379

[8] Kamso J, Mvika ES, Ota MO, Okeibunor J, Mkanda P, Mihigo R The contribution of the polio eradication initiative to narrowing the gaps in the health workforce in the African Region. Vaccine 2016; 34:5150–4. 27395564 10.1016/j.vaccine.2016.05.061

[9] Boston Consulting Group. Polio funded personnel’s involvement in routine immunization and broader immunization goals. Final report. Boston: Boston Consulting Group, 2015.

[10] World Health Organization. Immunization data: WUENIC DTP3 coverage. http://apps.who.int/immunization_monitoring/globalsummary/timeseries/tswucoveragedtp3.html Accessed 21 October 2016.

